# Preventive health resource allocation decision-making processes and the use of economic evidence in an Australian state government—A mixed methods study

**DOI:** 10.1371/journal.pone.0274869

**Published:** 2022-09-19

**Authors:** Jaithri Ananthapavan, Gary Sacks, Marj Moodie, Phuong Nguyen, Rob Carter

**Affiliations:** 1 Deakin University, Geelong, Australia; 2 Deakin Health Economics, School of Health and Social Development, Institute for Health Transformation, Geelong, Australia; 3 Global Centre for Preventive Health and Nutrition, School of Health and Social Development, Institute for Health Transformation, Geelong, Australia; PLOS ONE, UNITED KINGDOM

## Abstract

**Context:**

Recommended best practice for resource allocation decisions by governments include a stepwise process guided by economic evidence. However, the use of economic evidence in preventive health decision-making, which often impacts on multiple sectors of government, is under-researched. This study aimed to explore the resource allocation decision-making processes for preventive health interventions in the New South Wales (NSW) Government in Australia, and specifically examined the barriers and facilitators to the use of economic evidence from the perspective of multiple government departments.

**Methods:**

This mixed methods study was conducted using semi-structured interviews with NSW Treasury representatives (n = 4), a focus group of NSW Ministry of Health representatives (n = 9), and a quantitative questionnaire of all participants. The schedule for the interviews and focus group was based on resource allocation guidance documents from Australian government agencies. Deductive content analysis was undertaken, guided by the Multiple Streams Framework.

**Findings:**

NSW Treasury participants believed that decision-making processes where economic efficiency was the key guiding principle was the ideal approach. However, the NSW Ministry of Health participants identified that for preventive health decision-making, economic evidence was not used to inform their own choices but was typically only used to convince other agencies of the merits of proposed initiatives when seeking approval. The key barriers to the use of economic evidence were the lack of capacity within the NSW Ministry of Health to understand and undertake economic evaluations; a lack of collaboration between NSW Treasury and preventive health decision-makers within the NSW Ministry of Health; and deficient processes and governance mechanisms that do not facilitate or incentivise effective inter-sectoral decision-making.

**Conclusions:**

Institutional structures for resource allocation decision-making regarding preventive health result in processes that contrast with best practice recommendations. The multiple challenges to collaborative decision-making across agencies require organisational change to promote a whole-of-government approach.

## 1. Introduction

Modifiable risk factors are the leading cause of disease burden worldwide [1]. In Australia, one-third of disease burden is attributed to modifiable risk factors; with the risk factors attributable for the most burden being tobacco use, overweight and obesity, and dietary risks [2]. Greater action is therefore required to prevent the high prevalence of these three risk factors [3]. Effective action to change the environmental factors driving modifiable risk factors requires political support, whole-of-government collaboration and co-ordinated action across several government sectors [4–6]. To facilitate a whole-of-government approach to decision-making, policy-making should be supported not only by data related to the effectiveness of different interventions, but also the economic credentials of possible options for change [4,7]. Economic evaluations allow decision-makers to make an informed judgement on the value for money of different policy options based on the incremental cost of implementing the intervention, relative to the foregone benefit of maintaining the status quo [8].

Economic evaluation evidence has been recognised internationally by various national governments as being an important tool to promote consistency in decision-making [9]. Australian federal and state government central agencies (e.g. Treasury and the Department of Premier and Cabinet) recommend processes that line departments (e.g. health, transport and education departments) should follow when making resource allocation decisions, with evidence use embedded in these processes [10–12]. In line with recommendations from the European Union, the World Bank and many developed nations [9], Australian central agencies mandate the use of cost-benefit analysis (CBA) to inform all significant government actions and decisions related to projects, programs, policies and regulations [10,12].

Amongst the Organisation for Economic Co-operation and Development (OECD) countries, the use of economic evidence within national Health Technology Assessment processes have been established since the 1990s [13,14]. The importance of evidence, and more specifically economic evidence to improve the rationality of policy decision-making in other health-related decision-making contexts has recently gained greater recognition [15,16]. Several studies have examined the use of economic evidence and the enablers and barriers to its use in health policy decision-making [17–20]. Key findings are that despite decision-makers’ interest in and willingness to use economic evidence, in practice it is rarely influential in resource allocation decisions. Key barriers relate to both the accessibility and acceptability of economic evidence [14]. There is increasing recognition that the availability of high quality economic evidence alone is not sufficient to ensure rational decision-making, and the effective use of such evidence is largely dependent on the decision-making processes and the organisational, institutional, political and cultural dynamics of the healthcare system [13,21]. There continues to be limited evidence related to the determinants of the use of economic evidence in preventive health decision-making, with the majority of the evidence from the United Kingdom at the local government level. These studies report that the key barriers to the use of economic evidence in decision-making are organisational factors, such as skills and beliefs of senior staff and the local political agenda. Additional barriers relate to the limitations of current economic evaluation methodologies that are overly reliant on health outcomes [22–26]. A key recommendation for future research is to broaden the inquiry to better understand the complexities of sub national decision-making contexts and the full range of determinants of the use of economic evidence [14].

In Australia, the majority of the responsibility for preventive health is shared between federal and state governments [27]. Several Australian studies have identified key influences on policy-making processes for health interventions [28–30]. The availability of evidence has been identified as an important influence on policy decisions [31–33]. One study from the 1990s investigated the use of economic evidence in health policy decision-making within the Australian federal and NSW state governments and found that the need to make quick decisions, political factors, limited availability of data and lack of expertise were the key limitations in the use of economic evidence in decision-making [19]. However, resource allocation decision-making processes for preventive health and the way in which evidence, specifically economic evidence, is used in different stages of the policy process (e.g. development of potential policy actions, approval, implementation and evaluation) have not been documented. Despite several studies in the United Kingdom local government context, the organisational factors that impact preventive health decision-making in Australian state governments is likely different. Accordingly, there is limited contemporary evidence of the enablers and barriers to the use of economic evidence in Australian state government preventive health decision-making.

A previous descriptive study by Liu et al [34] explored the factors that drive decisions on evidence-based policy-making related to preventive health by Australian governments and private health insurers. A key finding was that perspectives varied between representatives from health and treasury departments. This highlights the need for further explanatory and interpretive research studies [35] that investigate these interactions and interpret the data from both perspectives. Such research could provide recommendations on how health and treasury could work together to establish a framework for the better use of economic evidence. A limitation of the Liu et al study [34] was that it was ‘a-theoretical’ (a theoretical framework was not used to explain findings). As a result, it arguably does not provide a complete understanding of study findings or a full exploration of the policy environment and its influence on the use of economic evidence in decision-making [31].

This study addresses current evidence gaps by adopting a well-established political science theoretical framework (Multiple Streams Framework (MSF)) to explore resource allocation decision-making processes for preventive health interventions and the barriers and facilitators to the use of economic evidence in the New South Wales (NSW) Government from the perspectives of both the NSW Ministry of Health and NSW Treasury.

## 2. Methods

### 2.1. Study design

A mixed methods approach was employed. Using the Leech and Onwuegbuzie 2009 [36] typology, the study utilised a Partially Mixed Concurrent Dominant Status Design. Dominant status was given to the qualitative data [37] collected from semi-structured in-depth interviews with participants from NSW Treasury (referred to as Treasury participants hereafter) and a focus group with participants from the NSW Ministry of Health (referred to as Health participants hereafter). Quantitative data were obtained from a questionnaire that provided supplementary data related to experience and familiarity with economic evaluation. The quantitative and qualitative data were collected concurrently using an ‘identical’ sampling strategy, and partially mixed and integrated during data analysis [36,38]. The primary purpose of the mixed methods design was to achieve complementarity, with the combination of results providing more meaning and validity to the study findings, whilst also enabling triangulation [39,40].

The interview schedule and the focus group discussion guide were based on document content analysis [41] of economic evaluation, CBA and decision-making process guidelines produced by Australian federal government departments [10,11,42,43], NSW central agencies [12,44], NSW line departments [45–48] and published examples of economic evaluations related to prevention [49,50]. Additional questions related to: i) interactions between the NSW Ministry of Health and central agencies (such as Treasury, and Premier and Cabinet); ii) interactions with other agencies outside the health sector (such as Transport for NSW and the NSW Department of Education) in relation to cross-sectoral policies; and iii) the barriers and facilitators to the use of economic evidence in decision-making processes. The interviews took place after the focus group, with initial impressions and issues raised from the focus group participants used to guide aspects of the interviews with participants from Treasury.

To account for the key influences on decision-making processes for policies related to preventive health, we drew on key concepts from the MSF. The MSF has been used extensively to analyse policy processes related to agenda setting over the last 30 years [51]. The framework emphasises the importance of values of the institution in the policy process [52]. The MSF assumes three independent streams (problem, policy and politics) come together at critical times (called “policy windows”) to create opportunities for policy action [51]. The ‘problem’ stream highlights the importance of problem framing and its impact on the potential government actions that can be ‘coupled’ to it. The ‘policy’ stream relates to how policy alternatives are generated, the criteria required for specific policies to survive, and emphasises how the structure and integration of policy communities impact the nature of the solutions. The ‘politics’ stream focuses on the dynamics of political actors and the political environment, and their impact on the adoption of policies [53,54]. The main assumptions of this framework and the key constructs of the three streams were used to understand the decision-making process within the NSW Ministry of Health with respect to preventive health interventions.

### 2.2. Data collection

#### 2.2.1. Study setting and participants

This study was undertaken within the NSW Treasury and the NSW Ministry of Health departments of the NSW Government in Australia. The researchers who conducted the interviews (JA) and the focus group (JA, RC and PN) and analysed the data (JA, MM and RC) were all experienced health economists. The study was undertaken with a constructivist epistemology and a critical realist perspective on mixed methods research. This epistemological position recognises that the role, relationship with participants, values, and the prior experience of the researchers shape all aspects of the study methods and findings. The critical realist perspective is consistent with both quantitative and qualitative methods and takes the view that they can work together to address the other’s limitations. It emphasises the importance of understanding divergent perspectives and alternative world views, and underscores the importance of context in the integration, interpretation, and generalisation of results. It is therefore particularly relevant for understanding decision-making processes and the importance of the context within which these processes occur [39,55,56].

Recruitment of participants was based on their experience and knowledge of i) how policy decisions are made within their own department; ii) the economic evidence that is considered when making policy decisions, and iii) how CBA can aid decision-making in the NSW Ministry of Health.

#### 2.2.2. Semi-structured interviews with NSW Treasury

The research contact within the NSW Treasury indicated that there were a limited number of employees within their department with relevant experience to inform the research. The research contact identified potential participants using purposive sampling. The snowball technique was then used to recruit additional participants. Of the seven potential participants invited, four participated in the study. Two invitees declined due to their limited knowledge of the subject area, and one was on leave. All participants were senior bureaucrats (i.e. principal analyst, associate director or director) across four areas of NSW Treasury. Interviews were undertaken face-to-face within NSW Treasury offices (1/4) or via telephone (3/4) between June and August 2018. The duration of the interviews ranged from 52 minutes to 68 minutes. See S1 Appendix for the interview schedule.

#### 2.2.3. Focus group with the NSW Ministry of Health

The research contact within the NSW Ministry of Health identified that i) the NSW Ministry of Health was a large agency; ii) the resource allocation decision-making processes may differ across different groups; and iii) there were not many staff within the department who had sound knowledge of economic analysis and its uses. The research team therefore decided that a focus group was the most appropriate format as the interaction between participants would be useful to develop a shared understanding. Purposive sampling was used to recruit senior bureaucrats (i.e. directors, principal policy analysts) and more junior staff members with relevant experience (i.e. research and evaluation officers) from various areas within the NSW Ministry of Health. The research contact also identified that decision-making processes often involved policy partners from the NSW Department of Premier and Cabinet (DPC) and therefore a representative from this department was also invited to the focus group. Eleven participants in total were invited to take part in the focus group. Five invitees were unable to attend, however they each recommended another participant within their respective areas to take their place at the focus group. Overall, there were 16 invitation letters sent, with 10 people from six different areas of the NSW Ministry of Health agreeing to participate. With one dropout on the day due to illness, the focus group consisted of nine participants. The focus group took place in June 2018 in the NSW Ministry of Health offices. The session was conducted over 2.5 hours with a 30-minute break. See S2 Appendix for the focus group discussion guide.

#### 2.2.4. Participant questionnaire

The participant questionnaire was supplementary to the qualitative data and was completed by all participants during the interviews and focus group. The questionnaire presented the opportunity for focus group participants to provide confidential data related to their perceived competence of themselves and their department to conduct economic evaluations, and the use of economic evidence and CBA in decision-making (S3 Appendix). Use of the same questionnaire among all participants allowed comparison between the Health and Treasury participant groups. The questionnaire consisted of 10 ordinal (Likert) scale questions with an odd numbered scale and two questions related to participant years of work experience in the NSW Government and qualifications or experience in health economics. For the focus group participants, the standard questionnaire was supplemented with four open-ended questions related to where participants would source assistance for economic evaluations both internal and external to the state health department. An odd numbered scale was chosen to reflect that the neutral option was a valid choice. Given that the questionnaire was administered during the interviews and focus group (concurrent study design), the number of questions was balanced with the time required to complete the questionnaire. Owing to the small cohort of potentially suitable participants for this study, formal testing of the validity and the reliability of the questionnaire with a sample representative of the research participants was not undertaken. The questionnaire was developed, reviewed, discussed, and revised by the authors.

### 2.3. Analysis

All interviews and the focus group were audio recorded and transcribed verbatim. Participants were provided with the relevant transcript and given the opportunity to check it for accuracy and clarify meaning of the information collected. Two participants made clarifications to the transcripts and the altered transcripts were used for data analysis.

The focus group was facilitated by JA. PN noted which participants made specific comments, and RC documented general impressions and emergent themes during the discussion. JA, PN and RC debriefed after the focus group and documented the additional field notes. JA conducted all the semi-structured interviews. Post interview, JA documented reflections and de-briefed with RC, PN and MM.

For the qualitative data (interviews and focus group), deductive content analysis was undertaken separately for each data set [57]. The coding frame was based on the interview schedule and focus group discussion guide, the key constructs from the MSF, and the CBA guidance documents produced by state and federal central agencies. Once each data set had been collected and initially analysed, the data related to each of the themes were mixed (partially mixed study design) and analysed again together to devise final themes [36]. The scale-questions in the questionnaire were analysed quantitatively and mixed with the initial findings from the qualitative data to assess whether the data was complementary or divergent to the qualitative analyses. Immersion in the data by JA as the developer of the interview schedule and focus group guides, focus group facilitator and interviewer shaped the initial coding framework. The coding framework was discussed with the research team prior to data analysis. Interpretations of the data were also discussed and checked with all authors to improve the credibility of the study findings. The findings and illustrative quotes generally represent the views of the majority of the focus group participants (group level data) and interviewees. It is made clear within the text when a view from only one participant is used.

The key findings, conceptualised using the MSF where relevant, are presented in Section 3 in four parts. Section 3.1 describes the resource allocation decision-making processes for preventive health programs and policies, followed by the barriers and facilitators of inter-sectoral decision-making (Section 3.2). Section 3.3 identifies the barriers to the use of economic evidence and Section 3.4 highlights the potential facilitators for the improved use of economic evidence.

The standards for reporting qualitative research (SRQR) checklist [58] has been completed and reported in S4 Appendix.

### 2.4. Ethics

Ethics approval for this study was obtained from the Deakin University Human Research Ethics Committee (reference number HEAG-H 180_2017) in January 2018.

## 3. Findings

### 3.1. Decision-making processes for preventive health and the use of economic evidence

**Fig 1:**
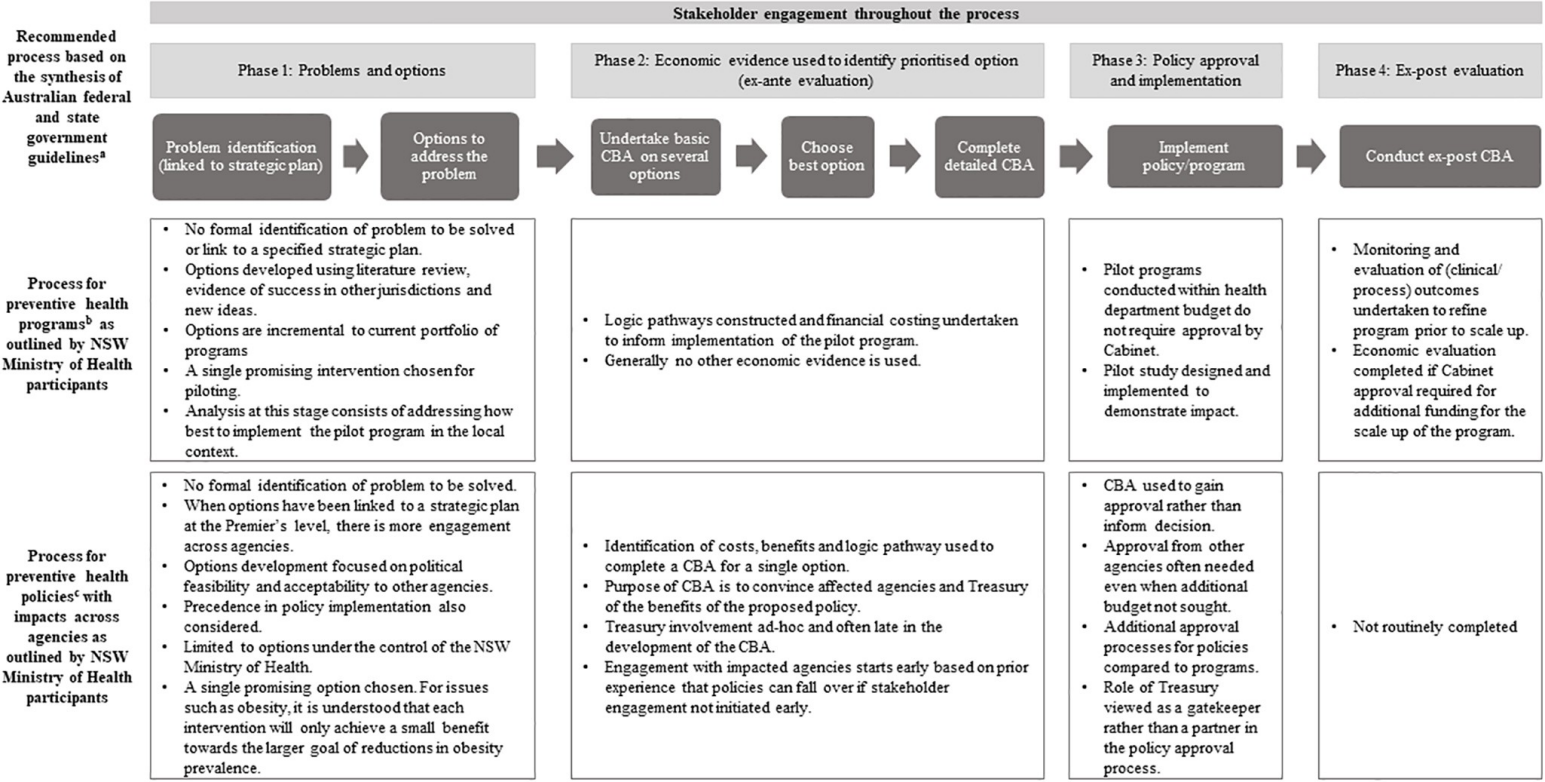
NSW Ministry of Health resource allocation process for preventive health mapped to the process recommended by official federal and state guidelines.

Guidelines from Australian federal and state governments recommend a clear, stepwise process for resource allocation decision-making where economic efficiency is the guiding principle and economic evidence is used at several stages [10,12,46,48]. A synthesis of these guidelines found that the general recommended process starts with problem identification and the specification of options for change, followed by the conduct of brief ex-ante economic appraisal on several options to choose the most efficient policy option(s), undertaking a detailed ex-ante CBA of the preferred option(s), and finally an ex-post analysis after policy implementation. Stakeholder engagement is recommended throughout the decision-making process (Fig 1).

All Treasury participants shared the view that decision-making with respect to prioritising programs and policies within the NSW Ministry of Health should follow this process. They agreed that resource allocation decisions should be informed by economic evidence with CBA being the preferred tool to produce this evidence. Some Treasury participants expressed frustration that there is inadequate use of economic evidence in some areas within the NSW Ministry of Health.

*“Evaluation was not necessarily prioritized*. *This [CBA] is the practice*, *they [NSW Ministry of Health] should be embracing that*, *you know…*.*having evaluation front*, *centre and back of every step*.*” [Treasury 1]*

Health participants, however, noted that the process of deciding what to include in their portfolio of work for preventive health was not as linear as recommended by the guidelines. Participants made a distinction between decision-making processes for ‘program-based’ and ‘policy-based’ preventive health initiatives (Fig 1). Although a definition of programs and polices was not provided by Health participants, they tended to refer to ‘program-based’ initiatives as projects where specific services were delivered, whereas participants referred to policies as changes to current government practices and requirements that impact population health.

Generally, health initiatives that do not change the regulatory environment nor require additional funding (outside the health budget) do not require Cabinet approval. Health participants reported that programs usually start as pilot programs that are generally funded within the health budget and, therefore, the resource allocation decision was largely made within the NSW Ministry of Health. They indicated that, although programs were typically based on evidence of effectiveness, the use of economic evidence in determining the potential options for implementation was limited.

Participants reported that rigorous evaluation was routinely undertaken to ensure the desired clinical and process outcomes (behaviour change, health, reach, etc.) were achieved. Financial costing was undertaken to inform pilot program implementation; however, economic evaluation was not routinely completed and was limited to occasions when additional funding bids were made for program roll-out (scale-up) and therefore approval by central agencies was required.

*“…yes if an intervention works then we fund it*. *And I think we’ve got that sort of philosophy–[cost-effectiveness is] not used very much at all*, *no*.*” [Health 1]* [There were five additional participants who agreed with this statement]

Compared to programs, focus group participants reported that there was less freedom in developing policy options related to prevention as there was a greater need to consider political feasibility and acceptability at the outset. Health participants reported that policies that were politically sensitive, for example, food related policies, and those that had an impact on other agencies and government systems, attracted the attention of central agencies, even when additional budget was not sought. Accordingly, NSW Treasury and agencies from impacted sectors were involved at early stages of decision-making processes. Treasury participants articulated the process they used to determine their involvement in the decision-making process for preventive health interventions. The key criteria related to the size of the investment and whether additional funding was required; whether there were cross-agency implications; the risk and substance of the proposal; and whether it was related to a national strategy. The NSW DPC was also involved in assisting the NSW Ministry of Health with resource allocation decisions for smaller proposals.

Health participants acknowledged that economic evidence was largely used to convince other agencies of the merits of preferred interventions rather than as an aid to making decisions within the Ministry of Health related to which programs or policies were best suited to address specific preventive health issues.

*“…as [Health 1 named] says*, *most of the CBAs are used to justify a position” [Health 2]*

The MSF offers several insights into why preventive health decision-making processes deviate from the rational evidence-based approach outlined by guidance documents. The NSW Ministry of Health limit the number of options for consideration beyond the initial ‘problems and options’ stage of the decision-making process (Fig 1). Successful implementation of policies in other jurisdictions is considered an *external factor* in the MSF and is theorised to influence the ‘policy’ stream [59]. Health participants reported that policies that have been implemented in other countries or jurisdictions were frequently targeted as potential policy options because successful implementation in other contexts increased the legitimacy of the policy option in the local context. Another key assumption of the MSF is that policy-makers have *time constraints* and need to *“…strike when the iron is hot”* [54]. Cognitive constraints limit the ability of policy-makers to consider a wide array of issues simultaneously. This coupled with limited time to act may diminish the feasibility of formulating an exhaustive list of policy options for CBA and explain why policy proposals that progress past the ‘problems and options phase’ are limited to promising interventions that are politically feasible [54]. One of the constructs of the ‘politics’ stream of the MSF is the *national mood*, referring to how the majority of the population feel about a problem or potential solution [54]. Responding to changes in the national mood may further explain the urgency felt by Health participants to progress policies that have gained a level of public acceptance in other jurisdictions and may be mirrored by the NSW population.

*“So it’s evidence informed*, *but it’s also looking at where precedent is as well*, *so where this policy has been implemented elsewhere*. *And then I think there’s a whole section around feasibility which is political feasibility but also palatability I guess…do you agree*.*” [Health 3] “Absolutely” [Health 1]*

The MSF notion of *ambiguity* was highlighted within the context of food policy related decision-making, where participants indicated that the various stakeholders have varied ways of thinking about the same issue and therefore the information or evidence used to inform policy solutions are also varied. As a result of both *ambiguity* and *time constraints*, *problematic policy preferences* [54] mean that policy preferences are not logical or complete and therefore unlikely to align with rational decision-making processes.

### 3.2. Inter-sectoral decision-making processes for preventive health interventions

All participants reported that there were no clearly documented processes for engaging different agencies in the decision-making process for policies (for example, advertising restrictions of unhealthy food and school-based initiatives) that have inter-sectoral impacts. Health participants reported that there were several formal and informal forums to engage with other agencies and discuss policy proposals; however, both Treasury and Health participants felt that there were several barriers to whole-of-government decision-making. Funding silos, where budgets of line agencies are delineated from other agencies despite having to work together to achieve common goals, were highlighted as the biggest issue. Other hurdles identified by participants included: i) the need for proposals to be owned by a single agency, which complicated initiatives that required joint responsibility across agencies for the achievement of outcomes; ii) competing priorities across agencies and the need for leadership buy-in across multiple stakeholders; iii) the time required to consider cross-sectoral impacts and, therefore, corresponding delays in progressing the policy process; and iv) the lack of data to assess the varied potential impacts of proposed initiatives on different stakeholders.

*“I don’t think there is a mandated process for doing that [engaging other agencies] other than I guess doing stakeholder analysis…*. *I think the primary challenge can often be a budgetary one in that proposals*, *particularly in the health or the human services space*, *might require an increase in investment from one agency but the benefits accrue to another agency*. *Getting agreement on those kinds of things and getting your accounting treatments to not adversely impact agencies’ budgetary performances can be quite challenging…*.*” [Treasury 3]*

Many of the key difficulties identified by Health participants associated with inter-sectoral decision-making relate to key constructs in the MSF. Health participants commented that the evidence base supporting their preferred interventions was often not believed by the different agencies impacted by the intervention. Health participants provided an example of where they were trying to persuade agencies to implement a policy related to the food environment that was likely to have a small impact on food consumption. They indicated that the specific policy initiative was aimed at taking a ‘leadership position’ where, although it was recognised that the single policy was unlikely to make a significant impact on the specific preventive health problem—in this example, obesity—the policy would send an important message that the government was taking action on improving food environments. However, Health participants reported that representatives from other impacted sectors opposed the policy based on the belief that the single policy was unlikely to make a meaningful difference to obesity levels. The MSF emphasises the importance of the framing of the problem and its impact on the possible solutions that can be ‘coupled’ to it [60]. In this situation, the MSF highlights that re-framing the problem in a way that matched proposed solutions may have been useful.

Focus group participants reported that when proposed policies impact other agencies, the process of engagement resulted in modified alternatives to the original proposal (often with reduced scope) moving forward in the decision-making process. The MSF theorises that the structure and integration of the *policy community* impacts on the nature of the solutions that gain momentum in the policy process [54]. This explains the observation by Health participants that policies that are approved, for example in the area of obesity prevention are often small incremental changes to the food environment. These ‘gradualist’ policies described by the Health participants are likely the result of the large, less integrated policy communities involved in the decision-making process [53].

*“You put up all the pieces of advice you possibly can in your recommendations*, *a decision is made that reduces your scope*.*” [Health 4] “Yes*.*” [unknown] “And then you build your best advice on what you’re left with*.*” [Health 4]*

Within the ‘policy’ stream, the MSF defines specific criteria for the survival of policy options that are related to values, feasibility and financial implications [54]. This may explain the relative ease in implementing preventive health programs that can be financed within the NSW Ministry of Health budget, where the policy community is smaller and values are more likely to be aligned. Health participants reflected on one example where the costs of policy implementation fell on another agency, and, therefore, the CBA faced higher levels of scrutiny. Health participants perceived that this extensive scrutiny of the CBA was to provide a reason to reject the policy due to the adverse financial impact that the policy would have had on that other agency, without due consideration of the overall benefits to population health. Health participants also expressed frustration that the personal values of the various actors played out in how much scrutiny the CBA received and was often used as a reason to reject proposals that were contradictory to ideologies related to the role of government in preventive health.

*“So when the hearts and minds aren’t there*, *then that’s when the cost benefit discussion perhaps comes into being*. *That’s my experience*. *So if there’s a politically challenging area*, *like food often is and perhaps where tobacco was many years ago*, *then it does come into being… It [discussion of the policy proposal] is a personal discussion sometimes across agencies*, *isn’t it*, *about their personal belief system*.*” [Health 3]*

*“You could have the best CBA in the world*, *and often do and have done*, *and still it’s put to one side for completely other reasons*.*” [Health 4]*

In relation to the Cabinet decision-making process, it was difficult to get a clear response from Treasury participants related to how proposals from different government departments were compared and prioritised. Although it appeared that CBAs for proposals across different sectors were not directly compared, they were examined in relation to how each met the government’s policy priorities.

*“That’s a huge question*. *I actually don’t know if I can answer that frankly*. *So*, *what I will say is that governments are elected to make resource allocation decisions across portfolios*, *and so at some point they are going to have to make trade-offs*, *because they’ve got a budget constraint*, *so ultimately someone is making a call*. *Now*, *as to how you get to that point and what is informing that decision*, *it really is wide and varied*. *I would say there’s lots of room for improvement in that process*. *I would argue that we’re all striving for evidence-based resource allocation*, *but that we’re a way away from that*.*” [Treasury 2]*

*“I think a direct comparison wouldn’t be [made]*. *I mean in terms of evaluating business cases*, *[proposals] are going to be evaluated on their own merit but I think perhaps a better way to frame it is that the prioritisation would be set by government in terms of where its policy priorities were*. *I mean government has its priorities and Treasury has a role in supporting delivery of those through advising Cabinet and the ERC [Expenditure Review Committee] around which proposals are going to best meet those*.*” [Treasury 3]*

Several of the Treasury participants mentioned that moving towards outcomes-based funding (where budgets are determined by the outcomes achieved) could resolve some of the issues related to inter-sectoral decision-making. They reported that moving away from siloed budgets could encourage departments to work together to achieve better outcomes for the NSW population. Health participants suggested that strengthening and standardising the methodology and process for proposal appraisal could help trump ideological objections that currently influence the decision-making process.

### 3.3. Barriers to the use of economic evidence in decision-making for preventive health

#### 3.3.1. Collaboration with NSW Treasury

Participants indicated that there were several aspects of the relationship between certain areas of NSW Ministry of Health and NSW Treasury that posed a barrier to the effective use of economic evidence in preventive health decision-making. Although Treasury participants were very clear on the reasons and situations in which they got involved in the decision-making process (see section 2.1), Health participants reported that it was not clear which policies Treasury were interested in being involved in and indicated that there was ambiguity regarding the section of Treasury that should be engaged for various policies.

*“…*.*[interactions with Treasury are] pretty ad hoc I’d say*. *But you’ve [Health participant named] probably got a more formal relationship…*.*” [Health 1] “Treasury is very engaged in some parts of the system and not others*. *So it’s really inconsistent I think*.*” [Health 2] “I think that the relationship [with Treasury] or the bar [set for policy approval] might be different*, *depends on where the policy idea comes from as well*. *So I think there’s…*.*inconsistency in the relationship” [Health 1]* [Several participants agree].

There was agreement amongst Treasury participants that the best outcomes in relation to developing a high-quality CBA that can be used in the decision-making process by Cabinet and other agencies were most likely achieved if analysts within NSW Ministry of Health worked in partnership with NSW Treasury and engaged with them early in the policy development process. However, Health participants reported that engagement with NSW Treasury was complicated by the different engagement processes of the various sections in Treasury (for example Commercial groups, Economic Strategy, and Agency Budget and Policy) and the conflicting advice received from the different areas.

*“I would say early engagement with Treasury is key*, *particularly when you are hitting estimated total costs that are high and projects that are risky…*. *If it [CBA] is not compliant*, *we will probably mention that in our advice to the Minister just letting them know—well*, *ideally it never gets to that point because we’ve worked together constructively to get to a point where we’ve got something that as a collective government unit we take up and say this is the best evidence that we’ve got” [Treasury 2]*

There was a perception amongst Health participants that NSW Treasury was focused on just the one method of economic evaluation—CBA. Health participants felt that NSW Treasury did not fully appreciate the merits of the other methods commonly used in preventive health, such as cost-effectiveness and cost-utility analyses. Some Treasury interviewees reported being more open to other economic analysis methods, whereas other participants from Treasury believed that CBA was the best approach. The differences in terminology and language also seemed to impede sound communication between these departments.

*“A shared language is really important though…*, *if you don’t talk the same language as Transport and Treasury*, *they won’t even consider what you calculate as the health benefits for that particular intervention*. *So I think agreement on that would be helpful*.*” [Health 5]* [Three other participants agreed].

The issues highlighted by the Health participants regarding a lack of clarity on when to involve Treasury and which parts of Treasury to involve relate to the MSF concept of *unclear technology* where individuals are unclear on how the processes within their agency impact the overall organisation, and are unclear of procedures outside their specific area [54]. This could be exacerbated by the MSF assumption of *fluid participation* [54] where Treasury staff turnover (evidenced by limited years of experience working within the NSW Government, see questionnaire results, S5 Appendix) results in the loss of institutional knowledge and changes to engagement processes with line departments.

Both Treasury and Health participants reported a lack of collaboration between the different departments, resulting in suboptimal working relationships that detracted from the mutual goal of improved outcomes for their constituents. As theorised in the MSF, Health participants believed that, similar to situations when engaging with other sectors (see section 3.2), when a policy was contrary to the value system of Treasury staff, there were additional hurdles for the preventive health policy before it could be approved.

The lack of effective communication between the NSW Ministry of Health and NSW Treasury was further highlighted in the questionnaire results (see S5 Appendix). Questions related to participants’ familiarity with various NSW Government economic evaluation guidelines revealed that both Treasury and Health participants were not familiar with many of the documents that outlined relevant policies and procedures. NSW Treasury guidelines on CBA [12] were familiar to all Treasury participants; however, only three Health participants indicated that they had used this document. NSW Treasury participants were unfamiliar with the NSW Ministry of Health guide to commissioning economic evaluations [45], while only a limited number of Health participants (2/9) indicated that they had used their own department’s economic evaluation commissioning guidance. The Health Infrastructure group within the NSW Ministry of Health had several guidance documents on CBA [46,47], however, only two Health participants were aware of these. This lack of familiarity with relevant documents revealed that there was a need for better dissemination, familiarisation and training related to the use of guidance documents between and within NSW Treasury and NSW Ministry of Health.

#### 3.3.2. Technical issues related to economic evaluation

Health participants reported that the complexity of preventive health intervention logic pathways, the associated assumptions, and the lack of data to support the assumptions were all barriers to the use of economic evidence. Health participants believed that other agencies did not trust the results of CBAs related to preventive health intervention. Several issues that contributed to the technical challenges were identified. Firstly, political and jurisdictional limitations meant that, in practice, there were limited available options for preventive health initiatives. Accordingly, state-level preventive health interventions, for example in the area of healthy eating, were often restricted in scope to small changes to food environments in limited settings. Relatively small, short-term impacts on behaviour in limited settings can be difficult to quantify, and, due to the lack of data from long-term studies, estimates of intervention effect from short-term studies needed to be modelled over longer timeframes to demonstrate impacts on health. Furthermore, Health participants reported that the modelled health impact was questioned by other agencies either because they were not familiar with the modelling approaches, they believed the impacts could not be attributed to the specific policy intervention, or they believed there were likely to be compensatory behaviours if policies only applied to specific settings.

*“I think the intervention itself [presents challenges]*. *We haven’t talked too much about that but sometimes because our interventions are quite complex and they involve that program logic [in order to demonstrate long-term impact] because we’re giving advice perhaps and then you’ve got to follow that through or we’re changing an environment*. *You have to follow that through to what that means [for health in the longer term]*. *There’s all these assumptions you make on the way…*.*having data*. *I think that’s a key issue in prevention*, *is the lack of data in the areas here*.*” [Health 3] “Attribution [attributing impact to the policy]*.*” [Health 2] “I think that’s where–yes the whole attribution but also the assumptions as well*, *for a CBA*. *But I think you’re just open to scrutiny*.*” [Health 3] “And in fact it’s kind of easy to pick us off [dismiss the policy proposal] isn’t it*?*” [Health 1] “Really easy*.*” [Health 3]*

Treasury participants understood the difficulties in quantifying and valuing the impacts of preventive health interventions and acknowledged the difficulties in developing CBA when there is limited evidence. However, they indicated that they needed confidence in the economic results to ensure they provided sound advice to Cabinet ministers.

*“I think it can be sometimes hard to quantify the costs and the benefits*. *And it’s hard to get a realistic assumption sometimes of the costs*, *and there can be so many benefits*, *and so many benefits beyond the actual policy that they’re very difficult to quantify*.*” [Treasury 4]*

*“Of course*, *the caveat on that is that not all agencies are created equal with respect to the evidence base they’re working from…*.*I think Treasury at times could be accused of being very disciplined and potentially even harsh in the way that we assess some CBAs that sort of don’t have that 40 years of evidence sitting behind them [like the Transport department]*. *And we’re trying to be pragmatic I guess is what I’d say*. *Obviously*, *there is a balance because we can’t put stuff up to ministers and say this is the BCR [Benefit Cost Ratio] result of this when we actually aren’t confident in the evidence sitting underneath it*.*” [Treasury 2]*

### 3.4. Facilitators of the use of economic evidence in decision-making

#### 3.4.1. Capacity building in economic evaluations within NSW Ministry of Health

Building the capability and capacity within the preventive health department to undertake economic evaluations was highlighted by both Health and Treasury participants as the key enabler to the increased use of economic evidence in resource allocation decision-making. The current lack of skills in economic evaluation within the preventive health group was highlighted in the questionnaire results, with only one focus group participant reporting that they were able to complete an economic evaluation. Less than half of the focus group participants (4/9) reported that they believed their department had adequate capacity to commission an economic evaluation, and all focus group participants believed there was inadequate capacity within their department to undertake economic evaluations.

Health participants believed that by conducting more evaluations, they would slowly build the evidence, assumptions and standardised methodologies for CBA for different areas of prevention, resulting in more buy-in and credibility for preventive health CBA from other agencies and Treasury. These views were echoed by Treasury participants.

*“The key success factors to getting good CBAs out of departments are*, *one*, *you have a really strong economics unit in that department and usually led by a chief economist who really knows what they’re doing*. *That internal capacity cannot be underestimated*. *It’s critical to success*. *It’s probably the critical success factor…*.*” [Treasury 2]*

In addition to capacity building, Treasury participants advised that the NSW Ministry of Health could better communicate the economic case for preventive health interventions in terms of highlighting the need for the intervention, the benefits it was likely to produce, and how it aligned to the strategic priorities of the NSW Government.

*“I think there are other agencies that do a really good job of tying the narrative around business cases to the broader policy objectives of government*. *So it–that’s kind of the broader system narrative*, *it’s something that I think Health could do better in terms of helping people who aren’t part of the health system get a clear understanding how particular policies or where particular investment decisions fit in within the broader system*.*” [Treasury 3]*

There was a reflection from a senior member of NSW Ministry of Health acknowledging that internal budget allocation processes could be improved, and the use of economic evidence would increase rigour in preventive health decision-making. There was agreement amongst Health participants that the development of the evidence base, capability and capacity was a ‘journey’ and there was commitment amongst Health participants to start that process.

*“But yes*, *how do we bring in another element [economic evidence] in terms of rigour for that internal decision-making which is just about applying a quality improvement process I suppose for us*, *or another decision-making tool as part of our development*. *I think you’re right [Health participant named] that you don’t sort of get to the end*. *You sort of start somewhere and then you sort of use the tools and see where it takes you as well*. *So I think that for us might help*.*” [Health 1]*

#### 3.4.2. Enhanced role of Treasury

Treasury participants believed they had various roles in improving the resource allocation decision-making process across all agencies. Several Treasury participants believed that NSW Treasury should mandate the use of CBA in decision-making but also provide training and assistance to build the capability of line agencies to undertake the evaluations. One Treasury participant believed that a key enabler to building the evidence base was the conduct and documentation of ex-post evaluations to better inform future ex-ante evaluations. A repository of CBA inputs across agencies was also suggested as a key enabler to the data issues faced by agencies.

*“I think it needs to be twofold*. *One needs to be the mandates and the other one is the knowledge*, *the capacity*. *I think just with the mandate is not enough*, *just with the capacity things will not work*, *they need to be both*.*” [Treasury 1]*

#### 3.4.3. Learning from other areas in NSW Ministry of Health

Participants indicated that the Health Infrastructure group (who were responsible for the planning and development of health capital works) within the Ministry of Health had embraced CBA and the decision-making process that NSW Treasury recommends. This group had developed its own CBA guidelines that were aligned to Treasury CBA guidelines, but tailored to the needs of the investment decisions made by Health Infrastructure. Participants representing this group valued CBA as a decision-making tool. The key insights provided by these focus group participants were that changing the resource allocation decision-making processes within the department takes time. These participants reinforced the importance of having CBA inputs and methods supported by the best available evidence and going through a peer review process to gain credibility. The participants from the Health Infrastructure group also conveyed a different attitude towards NSW Treasury, who were viewed as partners in the decision-making process rather than gatekeepers to policy approval. Although other Health participants believed there were lessons that could be learned from Health Infrastructure, they also believed that preventive health was a more complex area and therefore the ‘journey’ towards the better use of economic evidence in decision-making may be more complex.

## 4. Discussion

This mixed methods study investigated the resource allocation decision-making processes for preventive health interventions within the NSW Government. It explored different perspectives on the barriers and enablers to effective resource allocation decision-making, specifically for proposed preventive health interventions that have cross-sectoral impacts, and the use of economic evidence in the process.

The decision-making process recommended by Australian federal and state governments in their guidance documents [10–12,44], where economic evidence is used throughout the process, does not reflect the processes applied within the NSW Ministry of Health for preventive health interventions, where economic evidence was largely used only when needed to convince central or other agencies of the merits of an initiative prior to policy adoption. The mapped decision-making process showed the limited use of efficiency as a principle guiding the selection of programs and policies. The use of evidence as part of resource allocation decisions were largely related to evidence of effectiveness and evidence of successful implementation of interventions in other jurisdictions.

For preventive health programs, the decision context typically involved a smaller policy community (located within the NSW Ministry of Health) that allowed new ideas to be considered and implemented relatively quickly. Decision-making typically involved the selection of a single promising intervention in a particular intervention area. The assessment of acceptability was inter-connected with the specification of the intervention, with a focus on interventions that had gained legitimacy through implementation in other jurisdictions. This is supported by international evidence where even when the policy problem involved the need for a rational priority-setting process within a relatively small policy community (healthcare organisation in Canada), a comprehensive assessment of alternative approaches was not undertaken, but rather the more pragmatic option of Program Budgeting and Marginal Analysis (PBMA) was chosen as the alternative approach to the status quo, based on the experience of other similar organisations [61].

In the NSW context, there may be various explanations why economic evidence was not widely used. Firstly, the NSW Ministry of Health had a larger degree of control over decision-making [62], and economic evidence was not required if the program was funded within the existing NSW Ministry of Health budget. Secondly, when making decisions related to preventive health programs, there was an organisational culture where clinical efficacy and effectiveness were valued more as an aid to decision-making than economic evidence. Thirdly, the lack of capacity and expertise in health economics may have resulted in the benefits of using economic evidence not being well understood. The political nature of many preventive health policies [31,63] meant that a larger policy community was involved. This larger group of stakeholders resulted in policy ideas being debated, modified and rejected, with only policy options that had been adequately *softened* rising up as a potential solution [51]. The resulting policy options were likely to represent small, incremental changes to the status quo [53]. This was likely to have a flow-on effect where the recommended policy was only likely to make a small incremental change in population health, and, therefore, the policy’s economic credentials were less convincing. An early investigation into the use of economic evidence in health decision-making within Australian governments echoed some of our findings showing that key aspects of the decision-making process, including the need to make decisions quickly, were key barriers to the use of economic evidence. Other barriers included: lack of expertise, perceived lack of credibility of economic evidence, primacy given to clinical evidence over other forms of evaluation and lack of data [19].

The policy options that gain legitimacy are theorised to be influenced more by the values and beliefs of the policy community and the financial implications on other agencies than the suitability of the policy option to address the policy problem [54,64]. This was a key finding in our study where Health participants reported that differences in ideology between agencies involved in the decision-making process heavily influenced the policy options considered and the scrutiny applied to the technical analyses of the policies. This finding is echoed in two studies related to the policy process involved in passing two obesity prevention initiatives by the Victorian state government [32,33]. Both studies found that the neoliberal ideology of the policy actors heavily shaped the nature of the passed legislation [32,33]. We also found that the assessment of efficiency (the economic costs and benefits of a program) was not independent of issues related to financing and how proposed policies impact the budgets of various agencies involved.

Differences in perspectives between government departments may not be limited to ideology, but also differences in ideals or principles around priority-setting. NSW Treasury guidelines revealed a preferred process that was more aligned to explicit priority-setting using technical approaches advocated by many economists who believe that implicit priority-setting methods result in inefficiency and, in many cases, inequity [65]. The ideals underlying priority-setting may explain why processes within Transport departments [48], for example, are more aligned to the approach recommended by Treasury despite facing similar decision-making issues as the Health department. Further research into the values related to priority-setting processes and notions of efficiency and financial impacts across different government departments underpinned by appropriate priority-setting theories is required.

The challenges faced by the NSW Ministry of Health in cross-sectoral decision-making are well documented as common barriers to inter-sectoral decision-making related to preventive health nationally, internationally and at all levels of governance. These include: i) the complexity of preventive health interventions; ii) siloed working practices with prioritisation of different outcomes; iii) lack of a common ‘language’; iv) varied standards for evidence; v) lack of time, resources and incentives; vi) longer time-horizons required to demonstrate benefit from preventive health policies, and related issues in demonstrating the economic feasibility of these interventions; and vii) cultural differences [22,66–68]. The international literature suggests that deficiencies in governance arrangements were a key factor limiting the mobilisation of all sectors to support health promoting strategies across society, and further theoretical and empirical research into inter-sectoral governance arrangements is recommended [69,70]. Our study found that within the NSW Government, apart from the opportunities for cross-sectoral meetings, there was little governance of whole-of-government decision-making processes. Therefore, the multitude of barriers to successful inter-sectoral decision-making were likely to remain unless process improvement and organisational change were prioritised at a high level of government. Although these limitations apply to all government departments, preventive health policies are particularly impacted given that many effective policies require collaboration with government departments other than health [4–6].

Experience of other Australian state governments and international efforts with joined-up government and Health in All Policies (HiAP) initiatives can provide guidance on the facilitators for effective inter-sectoral decision-making [66,71–73]. Successful whole-of-government decision-making is reported to require political leadership at a high level with delegated responsibility for the delivery of the goals of the cross-government initiative and the required changes to organisational structures, governance and accountability [66,71–74]. The required ‘structural architecture’ includes a political mandate for change; incentives and performance based outcomes that create a need for formal inter-sectoral relationships and committees; dedicated funding; a strategic focus on collaboration to produce mutually beneficial outcomes; and training and skill development [66,71–74]. Treasury participants from this study reported that outcome-based funding was a potential tool to breakdown siloed government decision-making. This approach has been used as a resource allocation tool aimed at providing financial incentives to produce better outcomes in the education sector in the United States [75] and the health sector in the United Kingdom [76]. However, before outcome-based funding can be used to drive whole-of-government decision-making, the appropriate outcomes will need to be defined and the required ‘structural architecture’ will need to be prioritised and implemented.

As reported in studies of the UK parliamentary system, Treasury departments have a uniquely powerful position over the development of policies of other departments [77]. Similarly, in Australian state governments, Treasury departments monitor and regulate public spending and input into cabinet decision-making. Therefore, it is vital that line agencies develop and maintain effective working relationships with Treasury. This study found that the relationship between preventive health policy-makers in the NSW Ministry of Health and NSW Treasury personnel was characterised by deficient communication and engagement processes. The lack of an effective working relationship between the departments was exacerbated by the varied roles of the different groups in NSW Treasury, making it challenging to navigate the system. Poor communication by both departments was also evident in the lack of effective dissemination of guidelines and tools available to inform the conduct and use of economic evidence in decision-making. NSW Treasury could take steps to improve this relationship by progressing the narrative that it is also focused on improved outcomes for the NSW population and better communicate the desired engagement processes between government agencies and the various groups in Treasury.

The various data sources in this mixed methods study (interviews, focus group and questionnaire) supported the finding that the lack of capacity within the NSW Ministry of Health to understand and undertake economic evaluations was a key barrier to the use of economic evidence in decision-making for preventive health interventions. This was highlighted in the questionnaire results where all Health participants agreed that there was inadequate capacity within the department to complete economic evaluations. Building capacity to understand health economics within the NSW Ministry of Health has been previously recommended [34]. However, in addition to building an understanding of the basics of health economics, this study found that there was a need for more advanced skills that could be fulfilled by a health economics group embedded within the NSW Ministry of Health. Having a group within NSW Ministry of Health who understand the specific issues related to evaluating preventive health interventions and being able to communicate with NSW Treasury personnel using a shared language would be a key facilitator of change. Given that this would likely require substantial additional funding, a fundamental shift in current decision-making mandates would likely be required for resources to be prioritised to this function. There was also a need for the development of capabilities within NSW Treasury to better understand the issues with estimating the benefits of preventive health interventions and the methodologies currently used. This shared understanding between departments could result in the development of methods that increase the acceptability and validity of preventive health evaluations and, therefore, its use in preventive health decision-making. Australia has a well-developed, nationally co-ordinated evaluation process to determine the cost-effectiveness of pharmaceuticals and medical devices, and a clear process for the use of this evidence in resource allocation decisions [42,78]. It has been suggested that developing an equivalent process for the assessment of preventive health interventions would enhance the funding of cost-effective preventive health policies and programs [79]. A national evidence-based preventive health evaluation process would also alleviate the need for individual state health departments to develop their current lack of capacity in economic evaluation.

The MSF enabled a more realistic assessment of the decision-making process and the use of economic evidence compared to the process outlined in guidance documents. The use of the key assumptions and constructs of the MSF (*ambiguity*, *time constraints*, *problematic policy preferences*, *unclear technology*, *fluid participation and stream independence*) in analysing the results of this study helped to contextualise the influences on decision-making processes related to preventive health. Constructs related to problem framing within the ‘problem’ stream, the characteristics of the policy community within the ‘policy’ stream and the concept of the national mood from the ‘politics’ stream were particularly useful in exploring the impact of the policy environment on decision-making processes. However, MSF concepts related to the coming together of the three streams and the role of *policy entrepreneurs* in coupling the streams were not observed in our study. This is likely due to the focus of our research being general decision-making processes rather than the process for the enactment of a specific policy. The MSF is not designed to elucidate the role of institutional rules and norms (besides values) and how knowledge and capabilities of policy actors influence decision-making processes [54,64], and therefore our use of the MSF was limited in interpreting the facilitators and the barriers to the use of economic evidence in resource allocation decision-making. International studies have emphasised the importance of examining institutional features that pose as barriers or facilitators to the use of economic evidence in priority-setting decisions [15,80]. The critical realist perspective within the context of mixed methods research recognises that although theoretical frameworks can be useful in interpreting data, they are incomplete and do not offer an all-encompassing view of a phenomenon [55]. This stance underscores the relevance of discussing findings that are not supported by a particular theory, and is compatible with the use of several theories within mixed methods research [31]. Further research investigating the institutional characteristics of Australian state government departments, underpinned by appropriate institutional theories and theories of incrementalism in public policy-making, in conjunction with theories of the policy process, may provide a more complete understanding of the key influences on resource allocation decision-making and why they vary across government departments.

There are strengths and limitations of each of the methods used in this study. The use of multiple methods for data collection presented some opportunity for data triangulation and was likely to have improved the credibility of the study findings [39,57,81]. The interviews with NSW Treasury allowed in-depth exploration of issues and detailed understanding of context. A limitation of the Treasury interviews was the small sample size. We were only able to recruit four participants; however, our study contact within NSW Treasury indicated that there were a limited number of senior participants who would be appropriate for the study. During the snowballing recruitment process, the same participants were recommended for participation and, therefore, we are confident that the key perspectives from NSW Treasury were captured.

With respect to the focus group, the interaction between participants encouraged participants to reveal their perspectives by relating to and responding to the experiences of others in the group, thereby providing a rich data set. As a rule of thumb, it has been suggested that three to five focus groups should be conducted to ensure adequate coverage of the research topic and the range of perspectives [57]. In our study we conducted one focus group with nine participants. This reflects the small number of relevant participants working in the NSW Ministry of Health and the challenges of conducting a study with busy government bureaucrats. A key limitation of focus group methodology is its inability to address the relationship between sampling, representativeness and the prevalence of the views expressed by the group [57,82]. The differences between participants in specific social characteristics have been reported to impact the level of participation in focus groups [82]. Our focus group included NSW Ministry of Health staff at different levels. Often participants worked closely with other participants, and, in many cases, managers and their staff were participants. This impacted the nature of the interaction between participants and participation was slightly unbalanced, with senior participants being more vocal, and the more junior participants expressing agreement. The level of participation could reflect the degree of experience and expertise in relation to the research topic, however it could also reflect the differences in seniority between the participants. This could have been addressed by having separate focus groups based on level of experience, however the practicalities of working with a state government department meant that only one focus group was possible.

The questionnaire in this study was anonymised for the focus group participants, which helped to counteract the issues related to unbalanced participation. The questionnaire was embedded with the qualitative components of the study and, therefore, only the interview and focus group participants completed the questionnaire, resulting in a small sample size that is likely to limit generalisability. Although the questionnaire was limited in participant numbers, the method of administration had the advantage of high return rates and the presence of the researchers provided the opportunity for participants to clarify any questions, improving the quality of the responses [81]. A key limitation of the questionnaire was the lack of reliability and validity testing of the tool prior to administration.

This study identified key facilitators to the increased use of economic evidence in preventive health decision-making as: i) adequate capacity and capability to undertake economic evaluations within health departments, ii) developing and maintaining close working relationships between health and treasury departments, and iii) changes to institutional structures to promote a whole-of-government approach to decision-making. Although focused on preventive health decision-making, the lessons from this study may be equally relevant to other government departments and should be the subject of future analyses. Ideology, values, beliefs (including related to explicit priority-setting) and the size of the policy community were shown to have a substantial influence on the policy options considered by government departments. The role of Treasury and their influence on resource allocation process requirements and decisions are consistent across government sectors, however Treasury’s relationships and understanding of the policy context across government departments may vary. The challenges associated with cross-sectoral decision-making apply to all government departments, however it acutely impacts preventive health decision-making, given the cross-sectoral nature of many effective preventive health policies. These findings may be generalisable to other Australian jurisdictions and other countries with similar parliamentary systems, however the small sample size is a key limitation to generalisability. The use of a well-established policy theory and rich descriptions of the study context may assist with transferability, however will need to be further explored in varied contexts using larger sample sizes where possible.

## Conclusion

This mixed methods study investigated the relatively under-researched topic of resource allocation decision-making processes for preventive health in Australian state governments and the use of economic evidence within these processes. We identified that the institutional structures and the complexities of decision-making in the context of preventive health results in non-linear processes that are incongruent with central agency guidance. The study identified that key facilitators to enhance the use of efficiency as a principle in decision-making processes include better communication between NSW Treasury and preventive health policy-makers in the NSW Ministry of Health and a dedicated health economics group within the health department. This study draws attention to the challenges of inter-sectoral decision-making in regard to preventive health, where there is often a reliance on other sectors to work with the health department to effectively craft and implement policies. Until there are better inter-sectoral decision-making governance processes, these challenges are likely to remain.

## Supporting information

S1 Appendix NSW Treasury interview schedule. (DOCX)

S2 Appendix NSW Ministry of Health focus group discussion guide. (DOCX)

S3 Appendix Participant questionnaires. (DOCX)

S4 Appendix Standards for reporting qualitative research (SRQR) checklist. (DOCX)

S5 Appendix Questionnaire results. (DOCX)
