# Supplementary material for: Preventive health resource allocation decision-making processes and the use of economic evidence in an Australian state government—A mixed methods study
Source: PLoS One. 2022 Sep 19;17(9):e0274869. doi: 10.1371/journal.pone.0274869 (PMC9484643; doi:10.1371/journal.pone.0274869)
Supplement: S1 Appendix — (DOCX) [file pone.0274869.s001.docx]

**S1 Appendix: NSW Treasury interview schedule**

**Introduction**

Personal introductions and review of the plain language statement which covered the following:

- Background and aims of the study
- Interview duration and voluntary nature of participation
- Study process including the opportunity to review interview transcripts to check accuracy

1. **Demographics**
2. Please describe your role and your day-to-day activities.
3. How many years have you worked for NSW Treasury?
4. What types of economic evidence or economic techniques are you familiar with and most commonly used within NSW Treasury?
5. How many economists are there within NSW Treasury?
6. Follow up questions:
   1. Are you familiar with CBA as a technique for economic evaluation?

***Participant questionnaire part 1 (see Appendix 3)***

1. **Decision-making within NSW Health**

In this section, I am trying to get an understanding of the different levels of decision-making within NSW Treasury and the process of how NSW Health policies are reviewed by NSW Treasury.

1. NSW Treasury recommend that line agencies should consider integrating evaluations into the decision-making process. Can you describe how this should be done?
2. What type of health policies require NSW Treasury approval?
3. Can you please describe the approval process for a preventive health policy?
4. During the decision-making process, what are the interactions between NSW Health, NSW Treasury and the Cabinet?
5. At which points in the decision-making process is evidence of effectiveness of the proposed initiative required or used?
6. At which points in the decision-making process is economic evidence required or used?
7. What type of economic evidence is required or used at each point in the decision-making process?
8. When you require economic evidence, what is the process of sourcing this evidence?
9. From your perspective, what are the enablers that support the use of economic evidence in decision-making by line agencies such as NSW Health?
10. From your perspective, what are the barriers to using economic evidence in decision-making by line agencies such as NSW Health?
11. What are potential solutions to these barriers?
12. Follow up questions:
    1. What is the relationship between NSW Treasury and the following:
       - NSW Health
       - NSW Transport
13. **Inter-sectoral decision-making**
14. For programs or policies that either involve or impact more than one line agency, what is the process of identifying impacts on other sectors (e.g. nutrition policies in schools)?
15. Is there a formal process for line agencies to engage with other sectors for input into evaluations? Please describe.
16. In your opinion, how should inter-sectoral impacts be incorporated into the policy making process and its evaluation?
17. What are the key challenges in inter-sectoral decision-making?
18. **High level CBA**

| Cost benefit analysis (CBA) has the potential to include the full economic and social impact (societal welfare) of programs. All costs and benefits of a program are valued in monetary terms. *NSW Treasury’s* preferred approach to economic appraisal is CBA. |
| --- |

***Participant questionnaire part 2 (see Appendix 3)***

1. Are you familiar with the issues related to the use of CBA in the health sector?
2. What factors would enable/encourage greater use of CBA in decision-making by line agencies such as NSW Health?
