# Supplementary material for: Preventive health resource allocation decision-making processes and the use of economic evidence in an Australian state government—A mixed methods study
Source: PLoS One. 2022 Sep 19;17(9):e0274869. doi: 10.1371/journal.pone.0274869 (PMC9484643; doi:10.1371/journal.pone.0274869)
Supplement: S2 Appendix — (DOCX) [file pone.0274869.s002.docx]

**S2 Appendix: NSW Ministry of Health focus group discussion guide**

Power point slides were used to project the key questions during the focus group discussion

**Introduction**

Personal introductions and review of the plain language statement which covered the following:

- Background and aims of the study
- Focus group ground rules including maintaining confidentiality of the participants and views expressed
- Study process including the opportunity to review interview transcripts to check accuracy

1. **Experience in the room**
2. What type of work do you do?
   1. Policy
   2. Administration
   3. Research/evaluation
3. Who has had experience with economic evaluations?
4. Who has had experience with cost-benefit analyses?

***Participant questionnaire part 1 (see Appendix 3)***

1. **Decision-making within NSW Ministry of Health, NWS Treasury and the Cabinet process**
2. Describe the process of how a new preventive health policy/program/initiative is
   1. Formulated
   2. Approved
   3. Implemented
3. During the decision making process, what are the interactions between NSW Health, NSW Treasury and the Cabinet?
4. At which points in the decision making process is evidence of effectiveness of the proposed initiative required or used?
5. What type of economic evidence is required at each point in the decision making process? What is the process of sourcing this?
6. From your perspective and experience, what are the **enablers** that support the use of economic evidence in decision making?
7. From your perspective and experience, what are the **barriers** to using economic evidence in decision making?
8. What are potential **solutions** to these barriers?
9. **Inter-sectoral decision-making - examples of programs or policies that either involve or impact departments other than NSW Health**
10. Did you go through a process of identifying impacts on other sectors and what did this involve?
11. Is there a formal process for engaging other sectors for input into policies and evaluations?
12. What are the opportunities for interaction between NSW Health and other Departments?
13. From your perspective and experience, how should inter-sectoral impacts be incorporated into the policy making process and economic evaluations?
14. What are the key challenges for inter-sectoral decision making?

***Participant questionnaire part 2 (see Appendix 3)***

1. **When should CBA be used in the health sector (preventive health)?**
2. What criteria should be used to determine whether an economic evaluation is required?
3. What criteria should be used to determine the evaluation technique?
   1. Should all proposed NSW Health programs (ex-ante) have CBA?
   2. Should some programs have cost-effectiveness or cost-utility analyses and some have CBA?
