# Supplementary material for: Preventive health resource allocation decision-making processes and the use of economic evidence in an Australian state government—A mixed methods study
Source: PLoS One. 2022 Sep 19;17(9):e0274869. doi: 10.1371/journal.pone.0274869 (PMC9484643; doi:10.1371/journal.pone.0274869)

**S3: Appendix: Participant questionnaires**

- 1. NSW Treasury participant questionnaire – Part 1


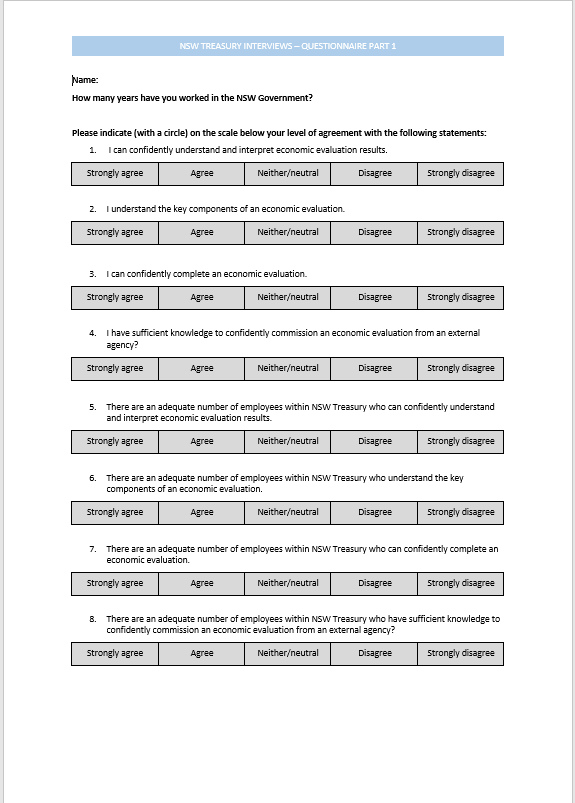


- 1. NSW Treasury participant questionnaire – Part 2

Front cover of the guideline documents (2a-2d) were shown to participants for Part 2 of the questionnaire.
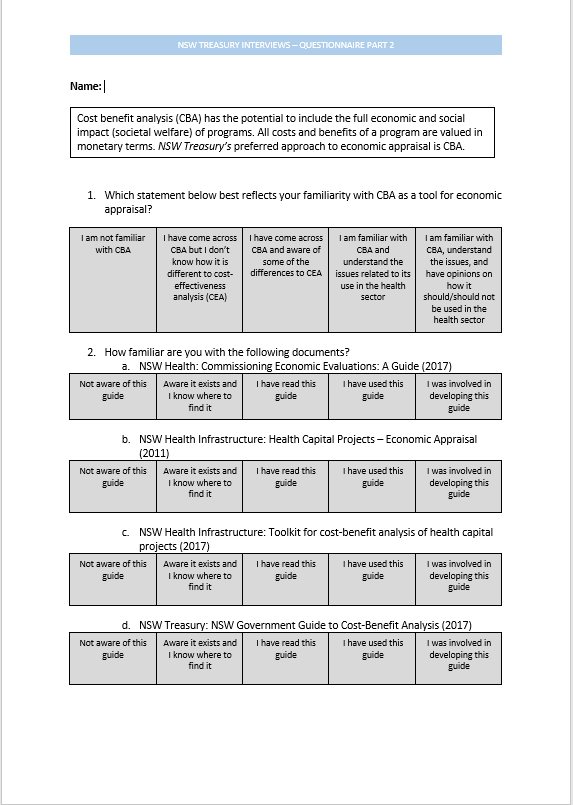


- 1. NSW Ministry of Health participant questionnaire – Part 1


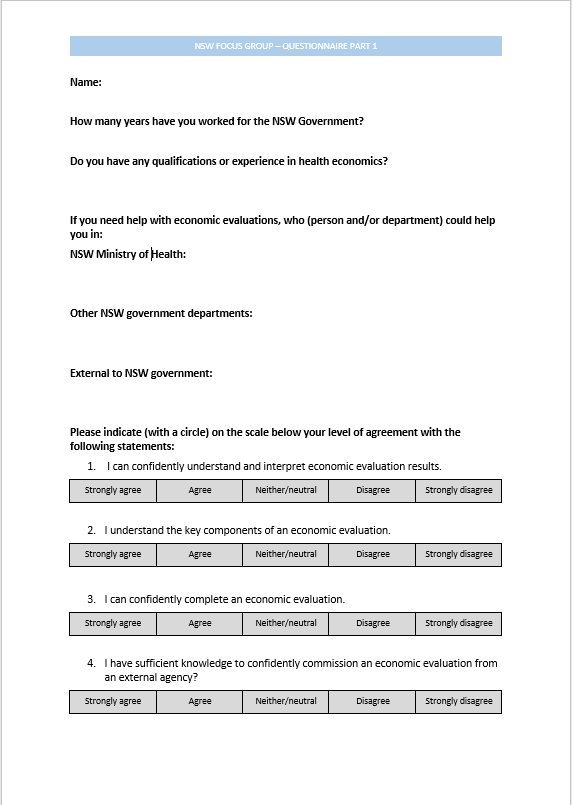


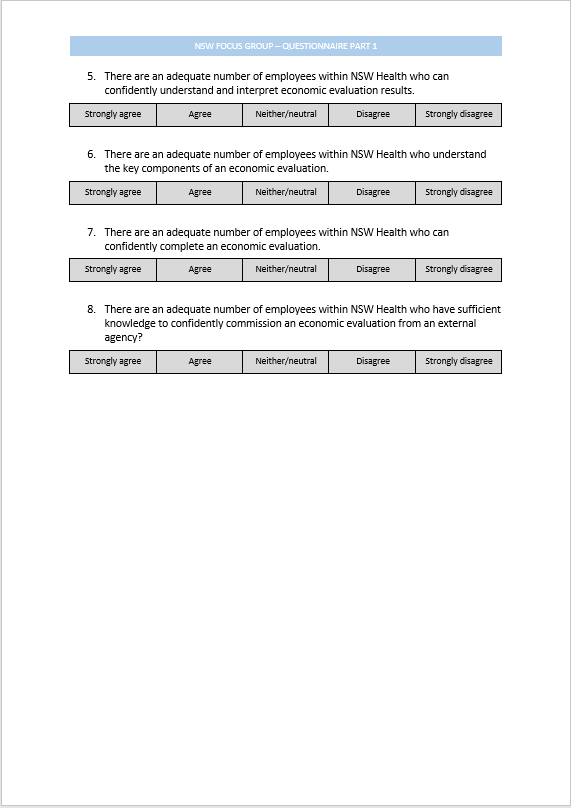


- 1. NSW Ministry of Health participant questionnaire – Part 2

Front cover of the guideline documents (2a-2d) were shown to participants for Part 2 of the questionnaire.


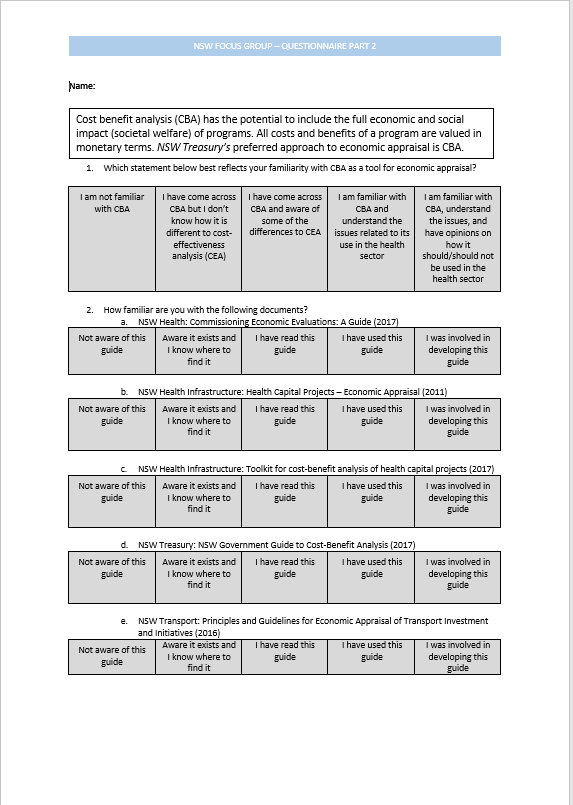

Supplement: S3 Appendix — (DOCX) [file pone.0274869.s003.docx]
