# Supplementary material for: Preventive health resource allocation decision-making processes and the use of economic evidence in an Australian state government—A mixed methods study
Source: PLoS One. 2022 Sep 19;17(9):e0274869. doi: 10.1371/journal.pone.0274869 (PMC9484643; doi:10.1371/journal.pone.0274869)
Supplement: S5 Appendix — (DOCX) [file pone.0274869.s005.docx]

**S5 Appendix: Questionnaire results**

|  | **Treasury** | **Health** |
| --- | --- | --- |
| Number of responses from participants | 4 | 9 |
| Years of experience within NSW Government | Mean = 2.8 (range: 2-3.5) years | Mean = 11.5 (range 1.5-30) years |
|  | **Number who scored 4 or 5^a^ (%)** | |
| I can confidently understand and interpret economic evaluation results | 3 (75%) | 6 (67%) |
| I understand the key components of an economic evaluation | 4 (100%) | 7 (78%) |
| I can confidently complete an economic evaluation | 2 (50%) | 1 (11%) |
| I have sufficient knowledge to confidently commission an economic evaluation from an external agency | 3 (75%) | 4 (44%) |
| There are an adequate number of employees within our department who can confidently understand and interpret economic evaluations | 4 (100%) | 2 (22%) |
| There are an adequate number of employees within our department who understand the key components of an economic evaluation | 4 (100%) | 2 (22%) |
| There are an adequate number of employees within our department who can confidently complete an economic evaluation | 3 (75%) | 0 (0%) |
| There are an adequate number of employees within our department who have sufficient knowledge to confidently commission an economic evaluation from an external agency | 4 (100%) | 2 (22%) |
|  | **Number who scored 4 or 5^b^ (%)** | |
| Which statement reflects your familiarity with cost-benefit analysis (CBA) as a tool for economic appraisal? | 4 (100%) | 2 (22%) |
|  | **Number who scored 4 or 5^c^ (%)** | |
| How familiar are you with the following documents? |  |  |
| NSW Ministry of Health, Centre for Epidemiology and Evidence: Commissioning Economic Evaluations: A Guide (2017) [1] | 0 (0%) | 2 (22%) |
| NSW Ministry of Health, Health Infrastructure: Health Capital Projects – Economic Appraisal (2011) [2] | 2 (50%) | 1 (11%) |
| NSW Ministry of Health, Health Infrastructure: Toolkit for cost-benefit analysis of health capital projects (2017) [3, 4] | 1 (25%) | 1 (11%) |
| NSW Treasury: NSW Government Guide to Cost-Benefit Analysis (2017) [5] | 4 (100%) | 3 (33%) |
| Transport for NSW: Principles and Guidelines for Economic Appraisal of Transport Investment and Initiatives (2016) [6] | 1 (25%) | 1 (11%) |
| **Tables Notes**  ^a^ 0 = No response; 1 = Strongly disagree; 2 = Disagree; 3 = Neutral; 4 = Agree; 5 = Strongly agree  ^b^ 0 = No response; 1 = I am not familiar with CBA; 2 = I have come across cost-benefit analysis (CBA) but I don’t know how it is different to cost-effectiveness analysis (CEA); 3 = I have come across CBA and aware of some of the differences to CEA; 4 = I am familiar with CBA and understand the issues related to its use in the health sector; 5 = I am familiar with CBA, understand the issues, and have opinions on how it should/should not be used in the health sector.  ^c^ 0 = No response; 1 = Not aware of this guide; 2 = Aware it exists and I know where to find it; 3 = I have read this guide; 4 = I have used this guide; 5 = I was involved in developing this guide. | | |

**References**

1. Centre for Epidemiology and Evidence. Commissioning Economic Evaluations: A Guide. In: NSW Ministry of Health, editor. Sydney: NSW Government; 2017.

2. Health Infrastructure. Capital Projects - Economic Appraisal. In: NSW Ministry of Health, editor. Syndey: NSW Government; 2011.

3. Health Infrastructure. Toolkit for cost-benefit analysis of health capital projects. In: Health Infrastructure, editor. Sydney: NSW Government; 2017.

4. Health Infrastructure. Toolkit for cost-benefit analysis of health capital projects. In: Health Infrastructure, editor. Sydney: NSW Government; 2017.

5. NSW Treasury. NSW Government Guide to Cost-Benefit Analysis. In: The Treasury, editor. Sydney, NSW: NSW Government,; 2017.

6. Transport for NSW. Principles and Guidelines for Economic Appraisal of Transport Investment and Initiatives: Transport Economic Appraisal Guidelines. In: Transport for NSW, editor. Sydney: NSW Government; 2016.
